# Comparative Study of Flax and Pineapple Leaf Fiber Reinforced Poly(butylene succinate): Effect of Fiber Content on Mechanical Properties

**DOI:** 10.3390/polym15183691

**Published:** 2023-09-07

**Authors:** Taweechai Amornsakchai, Sorn Duangsuwan, Karine Mougin, Kheng Lim Goh

**Affiliations:** 1 Polymer Science and Technology Program, Department of Chemistry, Faculty of Science, Mahidol University, Phuttamonthon 4 Road, Salaya, Nakhon Pathom 73170, Thailand; 2 Center of Sustainable Energy and Green Materials, Faculty of Science, Mahidol University, Phuttamonthon 4 Road, Salaya, Nakhon Pathom 73170, Thailand; 3 TEAnity Team Co., Ltd., 40/494 Soi Navamintra 111, Khet Bueng Kum, Bangkok 10230, Thailand; 4 Institut de Science des Matériaux de Mulhouse, IS2M-CNRS-UHA, 15, Rue Jean Starcky, B.P.2488, 68057 Mulhouse, CEDEX, France; karine.mougin@uha.fr; 5 Mechanical Design and Manufacturing Engineering, Newcastle University in Singapore, 172A Ang Mo Kio Avenue 8 #05-01, SIT@NYP Building, Singapore 567739, Singapore; kheng-lim.goh@newcastel.ac.uk; 6 Faculty of Science, Agriculture & Engineering, Newcastle University, Newcastle upon Tyne NE1 7RU, UK

**Keywords:** pineapple leaf fiber, flax, poly(butylene succinate), unidirectional composites, microstructure, mechanical properties

## Abstract

In this study, we compare the reinforcing efficiency of pineapple leaf fiber (PALF) and cultivated flax fiber in unidirectional poly(butylene succinate) composites. Flax, known for robust mechanical properties, is contrasted with PALF, a less studied but potentially sustainable alternative. Short fibers (6 mm) were incorporated at 10 and 20% wt. levels. After two-roll mill mixing, uniaxially aligned prepreg sheets were compression molded into composites. At 10 wt.%, PALF and flax exhibited virtually the same stress–strain curve. Interestingly, PALF excelled at 20 wt.%, defying its inherently lower tensile properties compared to flax. PALF/PBS reached 70.7 MPa flexural strength, 2.0 GPa flexural modulus, and 107.3 °C heat distortion temperature. Comparable values for flax/PBS were 57.8 MPa, 1.7 GPa, and 103.7 °C. X-ray pole figures indicated similar matrix orientations in both composites. An analysis of extracted fibers revealed differences in breakage behavior. This study highlights the potential of PALF as a sustainable reinforcement option. Encouraging the use of PALF in high-performance bio-composites aligns with environmental goals.

## 1. Introduction

In recent decades, escalating global concern over climate change has prompted a significant shift towards sustainable and environmentally friendly practices across various industries. One of the pressing issues we face today is the increasing level of carbon dioxide (CO_2_) in the Earth’s atmosphere, contributing to the greenhouse effect and subsequent climate change. As scientists and researchers strive to combat this challenge, exploring alternative materials and manufacturing processes becomes crucial in reducing our reliance on petroleum-based products while simultaneously addressing CO_2_ emissions using different concepts such as carbon capture utilization and storage (CCUS) using expensive modern technologies [1,2].

Plants, through the process of photosynthesis, have the remarkable ability to convert CO_2_ into organic compounds, effectively sequestering this greenhouse gas and mitigating its impact on the environment. Leveraging the inherent qualities of natural fibers in composite materials offers a promising avenue to not only decrease dependence on fossil fuel-derived resources, but also actively sequester CO_2_. By incorporating these natural fibers into polymer matrices, composites can be fabricated with improved mechanical properties [3,4,5] while simultaneously contributing to carbon capture and reduced environmental impact [6], similar to using wood, but much easier and with lower production cost. Although there are different types of natural fibers with a range of mechanical properties [4,7,8,9,10], perhaps it is fair to say that flax and hemp are the most successful commercial examples [11,12].

This study aims to conduct a comparative analysis between two types of natural fibers: flax and pineapple leaf fiber. Flax fiber, derived from the stem of the flax plant (Linum usitatissimum), has long been recognized for its mechanical strength and versatility [13,14]. On the other hand, pineapple leaf fiber, obtained from the waste of pineapple cultivation, has emerged as a potential alternative due to its abundance and renewability [15,16,17,18,19]. Although these two fibers have been studied for a long time, there is still some discrepancy in their reported mechanical properties (cf. Table 1 in [4], Table 2 in [15]). PALF has been used successfully in reinforcing different polymer matrices [20,21,22] despite the much lower than expected mechanical properties [23] compared to those of flax [24]. So far, there has been no direct comparison in the reinforcement efficiency of the two fibers, and thus this is the main objective of the present work. They are used to reinforce bio-based poly(butylene succinate) (PBS), which provokes an intensive interest among industry and researchers [25,26] and has been employed in automotive applications [27]. Here, we seek to investigate the influence of fiber content on the performance of the resulting composite materials.

Through this comparative study, we aim to contribute to the growing body of research dedicated to sustainable materials and their potential applications. By examining the mechanical properties of flax and pineapple leaf fiber composites, we can gain insights into their suitability for various engineering and manufacturing applications while also addressing the urgent need for carbon sequestration.

## 2. Materials and Methods

### 2.1. Materials

Poly (butylene succinate) (PBS, BioPBS FZ91PM/FZ91PB), produced from the polymerization of bio-based succinic acid and 1,4-butanediol, was used as the polymer matrix. The material was supplied by PTT MCC Biochem Company Limited (Bangkok, Thailand) and had a density of 1.26 g/cm^3^ and a melt index of 5 g/10 min (190 °C, 2.16 kg). Its reported molecular weight (*M*_w_) was approximately 170 kDa [28].

**Figure 1:**
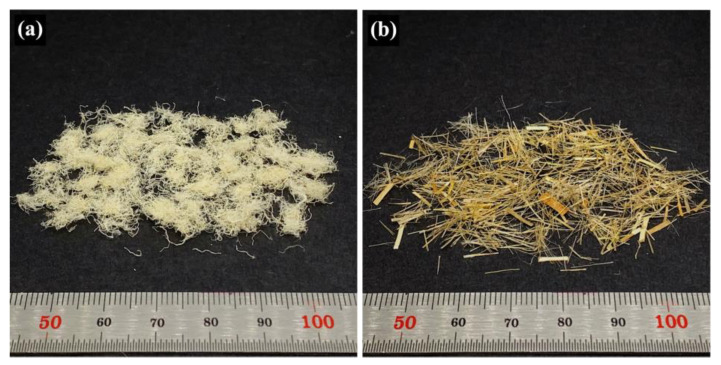
Photographs of (**a**) PALF and (**b**) Flax fibers.

Flax fiber (LINTEX, ~6 mm in length) was supplied by Dehondt Composites (Port-Jérôme-sur-Seine, France). According to the Alliance for European Flax-Linen and Hemp, the European flax is dew retted and mechanically scutched [29]. The fiber was supplied already cut to the specified calibrated length intended for composite reinforcement [30]. Pineapple leaf fibers (PALF, ~6 mm in length) were prepared from fresh pineapple leaves using the procedure presented in the literature [17]. Fresh pineapple leaves were collected from Bang Yang District, Phitsanulok Province, Thailand. The leaves were cut across their length into pieces 6 mm long, ground with a stone grinder, and dried to yield the whole ground leaf (WGL). The WGL was further processed by crushing it with a high-speed blender, followed by sieving to achieve the separation between the non-fibrous component and the PALF. The loose particulate non-fibrous component, with a particle size smaller than approximately 1 mm², was able to pass through the sieve. In contrast, the curly and entangled PALF remained on the sieve, highlighting its distinctive physical properties. For visual reference, photographs of both PALF and flax fibers are presented in Figure 1.

### 2.2. Composite Prepreg Preparation

Prior to the melt-mixing process, all materials were dried overnight in a hot air oven at 80 °C. The PBS pellets were then heated and melted on a two-roll mill (W100T, Dr. Collin GmbH, Maitenbeth, Germany) for 2 min at a speed of 30 rpm. The front and back roll temperatures were 125 °C and 100 °C, respectively. Subsequently, a predetermined amount of fiber (10 and 20 wt.% of total weight (PBS + fiber)) was gradually added over a period of 3 min. The mixing speed was then increased to 48 rpm, and the mixing continued for another 10 min to achieve a homogenous molten mixture.

**Figure 2:**
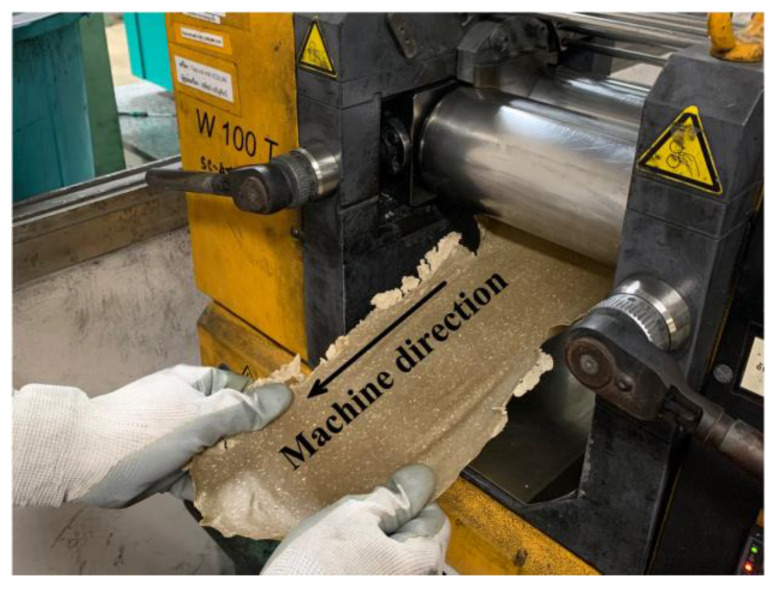
Fiber alignment on a two-roll mill during the uniaxial composite prepreg preparation.

The resulting molten mixture was carefully pulled out with slight stretching to maintain the alignment of the fiber parallel to the machine direction. It was then allowed to cool and solidify, forming prepreg, as illustrated in Figure 2. The composites were designated as 10PALF, 20PALF, 10Flax and 20Flax, denoting the respective content of the fiber in the composites.

### 2.3. Compressed Sheet Preparation

Composite sheets were prepared by stacking ten layers of prepreg between two flat metal sheets and a 3 mm spacer to prevent the excessive flow of the material and the disturbance of the fiber alignment. The stacked prepregs were preheated for 5 min under slight pressure. Then, they were pressed under a pressure of 1500 psi for 5 min, followed by cooling under the same pressure for 5 min. The compression molding was carried out at a temperature of 140 °C to destroy the matrix orientation and allow only fiber contribution to be observed [31].

### 2.4. Characterizations

#### 2.4.1. Fibers’ Chemical Composition

The chemical compositions of PALF and flax fibers were determined according to standard methods [32,33,34,35] through a certified local laboratory. Chemical composition is reported in terms of cellulose, holocellulose, acid-soluble lignin and acid-insoluble lignin.

The surface chemical compositions of PALF and flax fibers were observed using Fourier-transform infrared spectroscopy in an attenuated total reflectance mode (ATR-FTIR, Frontier, Perkin Elmer, Waltham, MA, USA). Spectra were recorded with 16 scans over the range of 4000 to 500 cm^−1^ with a resolution of 4 cm^−1^.

#### 2.4.2. X-ray Diffraction

X-ray diffraction patterns of the composites were recorded using an X-ray Diffractometer (XRD) (D8 DISCOVER, Bruker AXS GmbH, Karlsruhe, Germany) over the 2θ range between 5° and 80° with a step size of 0.02°. The X-ray wavelength was 1.54 Å (Ni-filtered CuK_α_). Pole figures for different samples were obtained with a cradle sample stage on the same machine. The data were analyzed with DEFFRAC.TEXTURE software (V4.1).

#### 2.4.3. Scanning Electron Microscopy (SEM)

Fibers’ shapes and sizes and the fractured surfaces of composites were observed using a scanning electron microscope (JSM-IT500, JEOL, Tokyo, Japan) with an accelerating voltage of 10 kV. Prior to observation, a thin layer of platinum was coated on the samples.

#### 2.4.4. Thermal Properties

The melting and crystallization behavior of the composites were determined with a differential scanning calorimeter (DSC) (Q200-RCS90, TA Instruments, New Castle, DE, USA). The samples were first heated from 25 to 200 °C, held for 5 min to completely melt all the crystals, cooled to −70 °C and then heated again to 200 °C. The heating and cooling rate was 10 °C/min under a nitrogen atmosphere. The positions of the melting peak (*T*_m_), enthalpy of fusion (Δ*H*_f_) and crystallization peak (*T*_c_) were determined for each sample using the instrument software. The degree of crystallinity (*X*_c_) was calculated using Equation (1).

(1) $$X_{c}=\left(\frac{∆H_{f}}{{∆H}_{f}^{0}\;(1-W_{f})}\right)\;\times \;100\%$$

where ${∆H}_{f}^{0}$ is the enthalpy of fusion for 100% crystalline PBS, which is taken as 110.3 J/g [36], and $W_{f}$ is the weight fraction of fiber in the composites.

In addition, the heat deflection temperature (HDT) was determined with a Gotech testing machine (HV-3000-P3C, Gotech Testing Machines Inc., Taichung City, Taiwan). The specimen sizes were 120 × 13 × 3 mm^3^. The test was performed following ASTM-D648 under the three-point bending mode with a span of 100 mm under a constant load of 0.455 MPa and a heating rate of 2 °C/min. HDT was determined as the temperature at which the specimen bends to 0.25 mm.

#### 2.4.5. Mechanical Properties

Flexural testing: The test was carried out on a universal testing machine (Instron 5569, Instron, High Wycombe, UK) at a crosshead speed of 5 mm/min, 1 kN of load cell and a support span length of 48 mm. The specimens were cut from compressed sheets into strips 12.7 mm wide with a long axis parallel to the machine direction. The average values of flexural strength and secant modulus at 1% stain from 5 specimens were reported.

Impact testing: The test was carried out on a pendulum impact testing machine (HIT5.5P, Zwick/Roell, Ulm, Germany) in Izod configuration. The impact specimens were cut from compressed sheets into strips 60 mm long and 12.7 mm wide. The samples were notched with a Zwick/Roell manual notch cutting machine. The notches were cut across the machine direction. Average values of 5 specimens were reported.

## 3. Results

### 3.1. PALF and Flax Characteristics

#### 3.1.1. Fiber Composition and Structure

**Figure 3:**
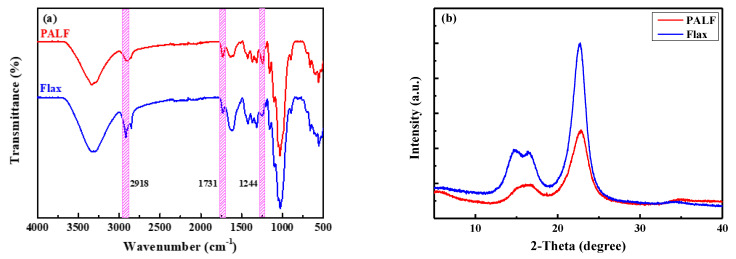
ATR-FTIR spectra (**a**) and XRD patterns (**b**) of PALF and flax fibers.

Table 1 displays the chemical composition of PALF and flax fiber. In general, the chemical composition of the two fibers is very similar. PALF has a slightly higher holocellulose content than flax fiber, while flax has about 10% greater cellulose content than PALF. PALF also has about 1.5 times higher acid-soluble lignin content than flax. The greater content of hemicellulose (the difference between holocellulose and cellulose) is reflected in the FTIR spectra shown in Figure 3a, in which the peaks at 1731 cm^−1^ and 1244 cm^−1^ correspond to the C=O stretching of hemicellulose and lignin and the C–O stretching in lignin, respectively [37,38]. The peak at 2918 cm^−1^ corresponds to the C–H stretching of methyl groups (–CH3) in both hemicellulose and cellulose [39].

The lower hemicellulose content in flax is a result of the enzymatic degradation of the binding material during dew retting [14]. For PALF, only mechanical force was used in the preparation of the fiber. Therefore, not much material was removed.

The crystalline structures of PALF and flax fibers were investigated using X-ray diffraction techniques. The diffraction patterns are shown in Figure 3b. Both fibers exhibited a similar characteristic pattern to cellulose Type I [40]. However, the patterns differed significantly in the resolution and sharpness of the peaks; flax fiber has much sharper peaks than PALF. This indicates that the crystalline structure in flax fiber is more perfect and possibly larger than in PALF. This difference could be the main reason for the higher mechanical performance of flax fiber.

#### 3.1.2. Scanning Electron Microscopy (SEM)

**Figure 4:**
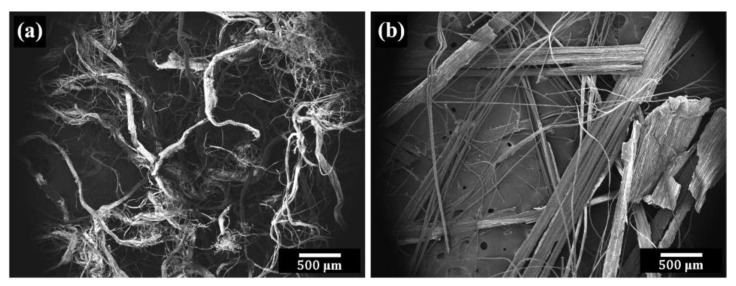
SEM micrographs of (**a**) PALF and (**b**) flax fibers.

Figure 4 compares the size and shape of the two fibers. PALF had both large bundles and fine elementary fibers. The fibers were not straight, but contained a lot of kinks. On the other hand, flax fibers were rather straight and had both isolated small fibers and bundles of fibers. Flax featured larger fiber bundles than PALF. Additionally, both PALF and flax fibers contained non-fibrous components. The variations in fiber size and shape can be attributed to the specific fiber preparation techniques employed.

#### 3.1.3. Prepreg Appearance

**Figure 5:**
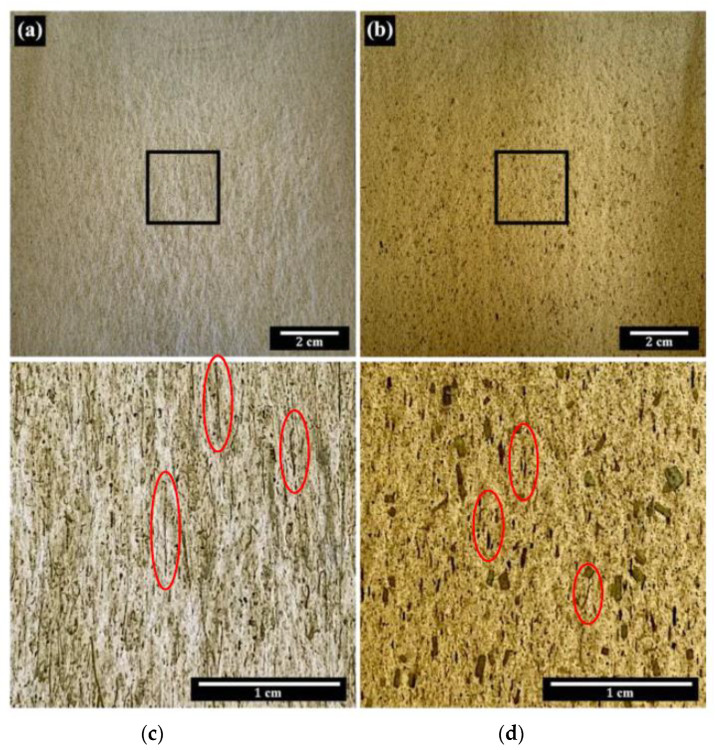
Optical images of composite prepregs: (**a**) PALF/PBS and (**b**) flax/PBS. Images (**c**) and (**d**) depict magnified views of the regions of interest indicated by squares in images (**a**) and (**b**), respectively. The red elongated circles highlight the alignment of fibers. Machine direction is vertical.

Figure 5 displays the photographs of PALF/PBS and flax/PBS prepregs. PALF/PBS has a pale color, while flax/PBS is brownish with a much greater number of dark spots of non-fibrous components. Both PALF and flax fibers appear evenly dispersed throughout the prepregs, indicating thorough mixing and alignment, as highlighted by the dark lines within elongated red circles.

### 3.2. Mechanical Properties of Composites

#### 3.2.1. Flexural Properties

**Figure 6:**
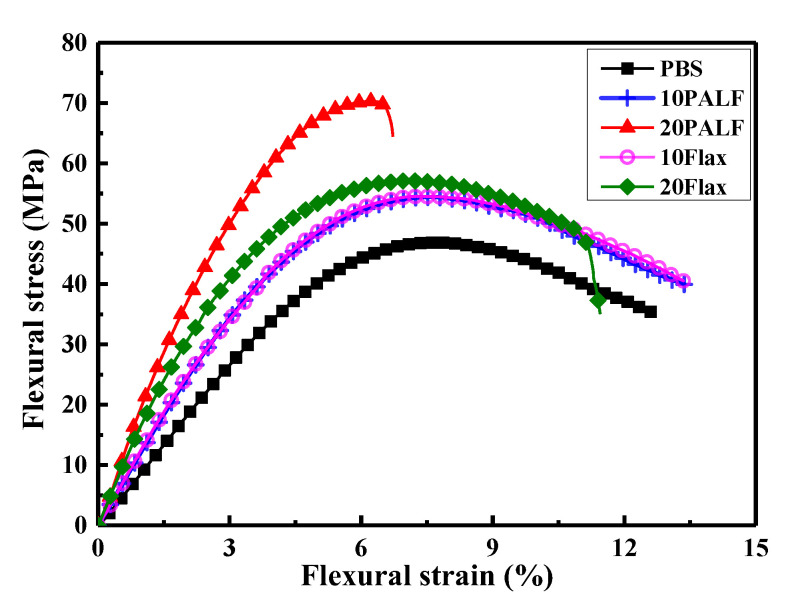
Representative flexural stress–strain curves of PALF/PBS and flax/PBS composites containing different fiber contents.

Figure 6 displays representative stress–strain curves of PALF/PBS and flax/PBS composites containing different fiber contents, and also that of PBS. With a fiber content of 10 wt.%, the stress at different strains increased over that of PBS throughout the whole range of strain. The composites had roughly similar failure strains to that of PBS. At 10 wt.% content, PALF/PBS and flax/PBS composites exhibited virtually the same behavior.

When the fiber content was increased to 20 wt.%, the stress for the flax/PBS composite increased slightly over that of 10 wt.%, and then the stress gradually decreased and the composite failed at a slightly lower strain than the composite with 10 wt.% fiber. The PALF/PBS composite with 20 wt.% fiber content exhibited a much-improved performance but failed at a much lower strain than the flax/PBS composite.

**Figure 7:**
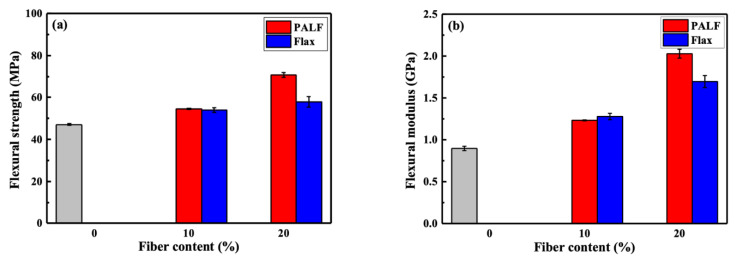
Flexural properties of PALF/PBS and flax/PBS composites containing different fiber contents, (**a**) flexural strength and (**b**) flexural modulus at 1% strain. Gray bar represents neat PBS.

The average values for the flexural strength and flexural modulus of these composites are shown in Figure 7. The average flexural strength increased from 47 MPa to approximately 54 MPa for both PALF/PBS and flax/PBS composites containing 10 wt.% fiber, and to 70.7 and 57.8 MPa for PALF/PBS and flax/PBS with 20 wt.% fiber, respectively. A similar trend was observed for the flexural modulus. The average flexural modulus increased from 0.90 GPa to 1.25 GPa for both PALF/PBS and flax/PBS composites containing 10 wt.% fiber, and to 2.03 and 1.70 GPa for PALF/PBS and flax/PBS with 20 wt.% fiber, respectively.

The above results clearly indicate a better reinforcement efficiency of PALF over that of flax, despite its inferior mechanical properties. Doubling the flax content from 10 wt.% to 20 wt.% caused the flexural modulus to increase by approximately 10%, but it caused only a marginal change in flexural strength. PALF, on the other hand, caused both the flexural modulus and flexural strength to increase by approximately 62% and 31%, respectively, under a similar change. The reasons for this will be addressed later.

#### 3.2.2. Impact Properties

**Figure 8:**
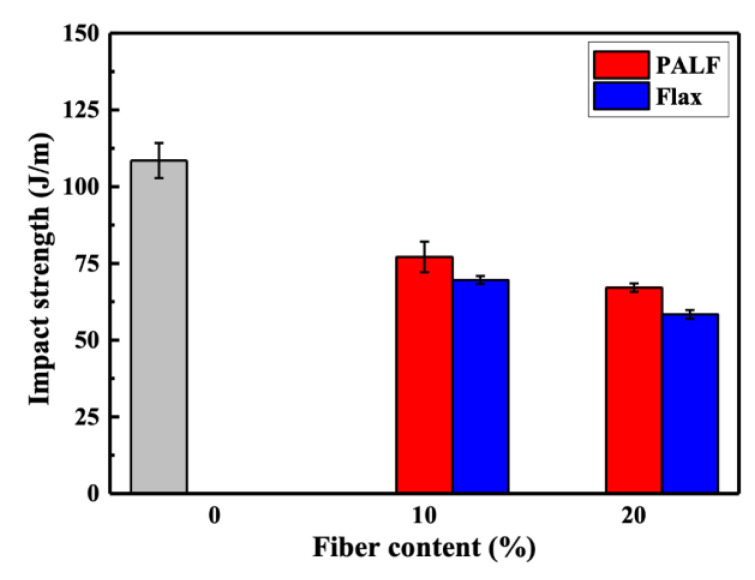
Impact properties of PALF/PBS and flax/PBS composites containing different fiber contents. Gray bar represents neat PBS.

Figure 8 displays the notched Izod impact strengths of PALF/PBS and flax/PBS composites containing different fiber contents. The introduction of 10 wt.% of fiber to PBS resulted in an impact strength reduction to approximately 70% and 64% of that of PBS for PALF and flax, respectively. With a further increase in fiber content to 20 wt.%, the impact strength dropped even further, to approximately 62% and 54% of that of PBS for PALF and flax, respectively. This indicates that PALF contributes to a smaller reduction in the impact strength of the composite compared to flax fibers. The decrease in impact strength in natural fiber-filled polymers is an anticipated outcome due to the increase in material stiffness and the presence of stress concentrators within [41,42,43]. A more detailed discussion on the reason for the comparatively smaller reduction in impact strength in the PALF system will follow.

### 3.3. Thermal Properties

#### 3.3.1. DSC

The melting and crystallization behavior of PBS in the composites is shown in Table 2. The presence of both PALF and flax fibers has a negligible effect on the melting temperature (*T*_m_), degree of crystallinity (*X*_c_), and crystallization temperature of PBS (*T*_c_). Thus, it may be stated that both PALF and flax fibers do not influence matrix crystallization, similar to the results observed in other systems [44,45].

#### 3.3.2. HDT

**Figure 9:**
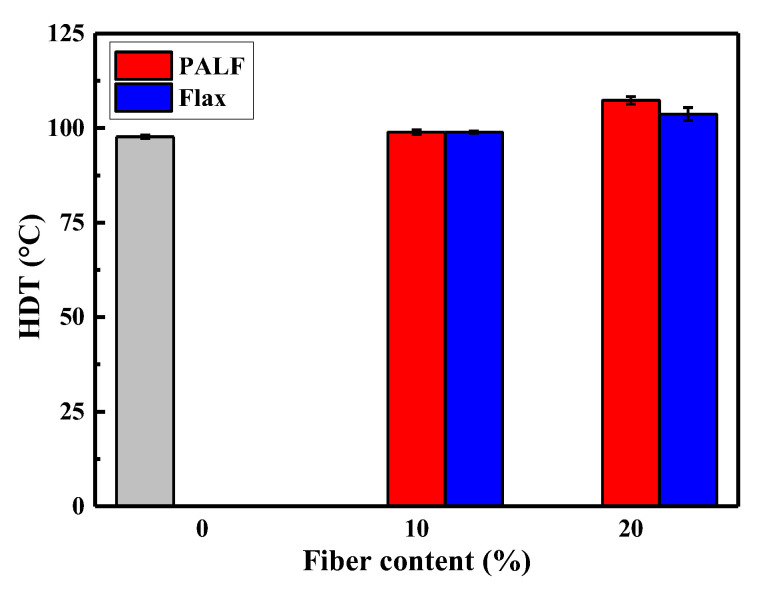
HDT of PALF and flax composites containing different fiber contents. Gray bar represents neat PBS.

Figure 9 displays the heat distortion temperature (HDT) of the composites along with the base PBS. At 10 wt.%, both PALF and flax had a negligible effect on HDT. However, when the fiber content was increased to 20 wt.%, PALF caused a larger increase than flax fiber, being approximately 10 °C and 6 °C higher than that of the base matrix. This is the consequence of the increase in flexural modulus of the respective materials.

### 3.4. Fracture Surfaces

**Figure 10:**
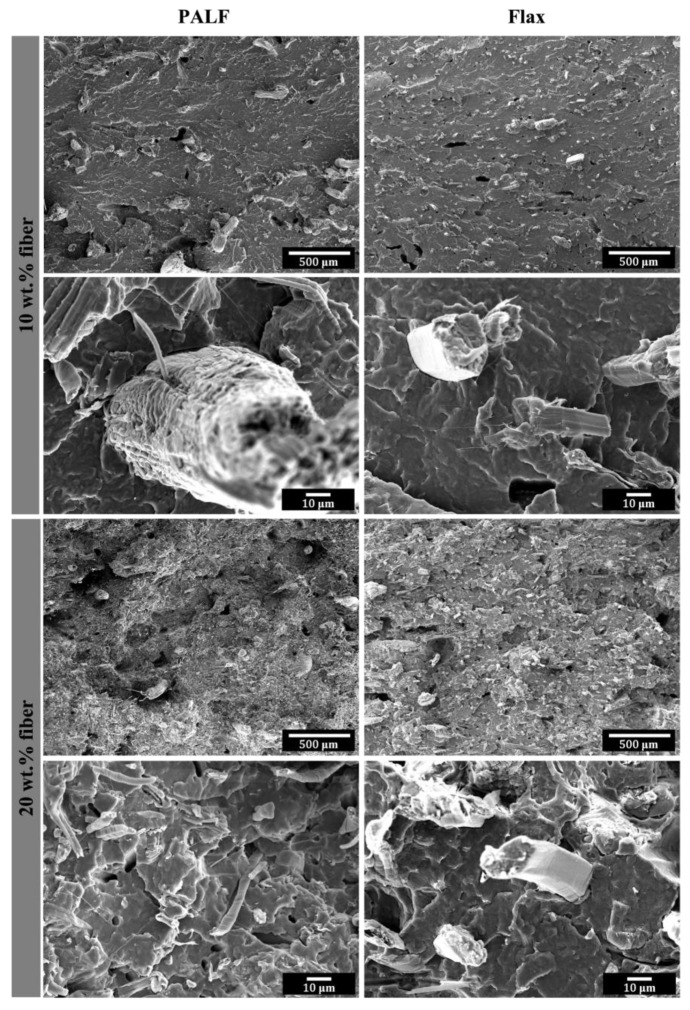
Impact fracture surfaces of PALF/PBS and flax/PBS composites containing different fiber contents.

Figure 10 shows the impact fracture surfaces of PALF/PBS and flax/PBS composites containing different fiber contents. Broken fibers are seen end-on, indicating a good alignment of the fibers along the machine direction (toward the observer). For 10 wt.% fiber, a larger number of fiber bundles can be seen in the PALF/PBS composite compared to the flax/PBS composite. When the fiber content was increased to 20 wt.%, a smaller number of large fiber bundles could be observed, indicating the breaking of large fiber bundles into finer elementary fibers. This phenomenon is likely due to the increase in the viscosity of the mixture (resulting from the higher fiber content), which facilitates higher stress transfer and thus breaks the bundles into finer elementary fibers.

## 4. Discussion

The nearly identical curves seen for 10 wt.% PALF/PBS and flax/PBS in Figure 6 signify a remarkable parity in reinforcing efficiency between the two types of fibers, even amidst their distinct mechanical properties. Intriguingly, at a higher fiber content of 20 wt.%, PALF exhibited significantly greater reinforcing efficiency than flax fiber. These findings merit a more in-depth examination.

It is known that for short fiber composites, the mechanical behavior of the composite is determined by several factors, including the mechanical properties of the reinforcing fiber, its orientation, the fiber aspect ratio, the fiber volume fraction, and the nature of the interface between the fiber and the matrix [46]. While we kept most starting parameters of the two types of fibers as close as possible, such as their length, amount, and mixing procedure, the only known parameter that was different was the mechanical properties of the fibers, with flax having much superior values. Surprisingly, this difference in mechanical properties alone does not fully explain the stark difference in reinforcing efficiency. Therefore, a deeper analysis of the internal structure of the composites, including matrix structure, matrix orientation, and fiber dimension, is required.

### 4.1. Matrix Orientation via Pole Figures

**Figure 11:**
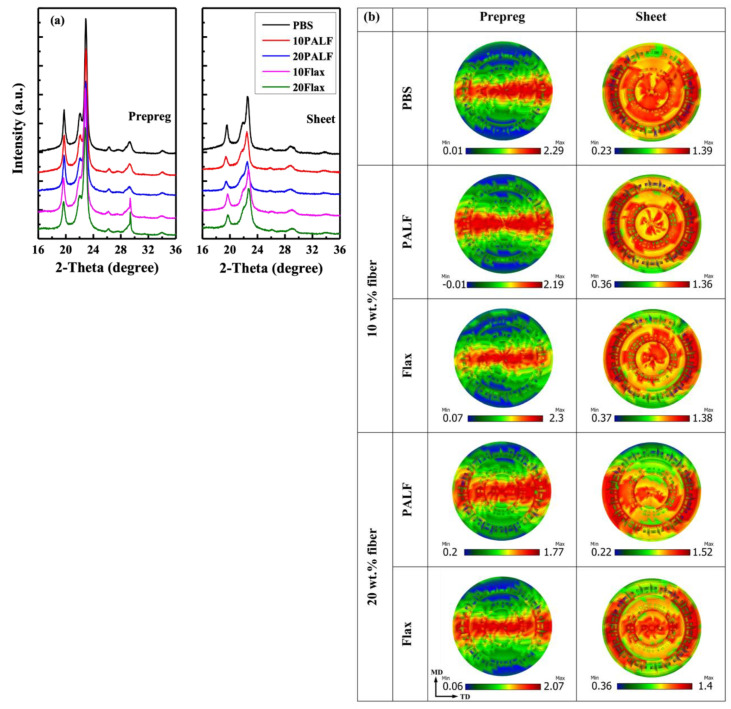
(**a**) XRD patterns of composite prepregs and sheets; (**b**) X-ray pole figures for (110) plane of PALF/PBS and flax/PBS composite prepregs and sheets compressed at 140 °C.

The production method for uniaxial prepreg employed in this work could lead to matrix orientation [31]. This had been destroyed during the compression molding by using a high compression molding temperature, as previously described. XRD was used to confirm this. Figure 11a displays the XRD patterns of composite prepregs and sheets. Prepregs display very strong intensity, while the sheets show much less intensity, indicating much relaxation of the polymer matrix. Pole figures for all samples were then determined using the most intense peak, at around 22.7°, which was associated with the (110) plane of PBS crystalline [47].

Pole figures for all samples for the (110) plane are shown in Figure 11b. It is clearly evident that the peak intensity of the prepregs is concentrated in the center, indicating a preferred matrix orientation in all prepregs. The presence of a high-intensity region supports the fact that the (110) reflection of the drawn PBS film lies on the equator [48]. However, when the sample was compressed at 140 °C, the previous orientation disappeared. These results are consistent with the previous XRD and DSC findings. Notably, with an increased fiber content in the sample compressed at 140 °C, a relatively weak molecular orientation can still be observed in the case of PALF/PBS. This suggests that the presence of fibers could slow down the relaxation of the matrix in the vicinity of the fiber, as suggested in the literature [31]. However, it could be assumed that such a marginal orientation of PBS in the PALF/PBS composite would play no role in enhancing the PALF/PBS composite over that of the flax/PBS composite (cf. Figure 7).

### 4.2. Reinforcing Fiber in the Matrix

**Figure 12:**
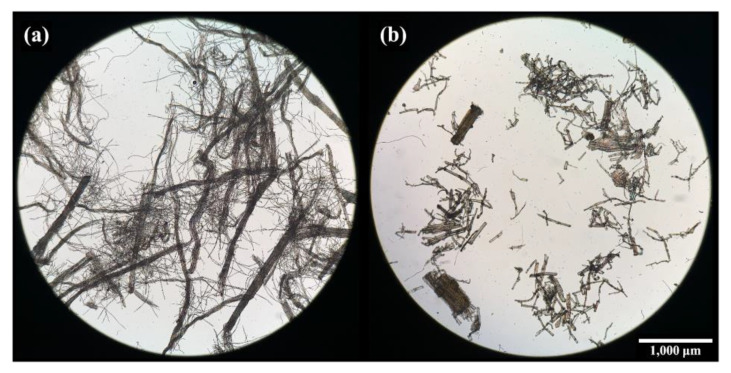
Low magnification optical micrographs of (**a**) PALF and (**b**) flax fibers after solvent extraction from their respective 20 wt.% composite prepregs.

It has been reported that fibers such as jute, flax [49], PALF [50], kenaf [51], poplar wood, radiata pine, and rice husk [52] can break down during incorporation into a polymer matrix, resulting in a lower reinforcing efficiency. To determine whether such a situation had occurred, the fibers were extracted from the composites using hot chloroform. Figure 12 displays optical images (Olympus BX51TRF, Olympus Optical Co. Ltd., Tokyo, Japan) of PALF and flax fibers that were extracted from PALF/PBS and flax/PBS composites with 20 wt.% fiber. It is clear that PALF remained long, while flax broke into very short pieces. In both cases, fine elementary fibers and large bundles can be seen. Thus, it is unquestionable that such fragmented flax fibers would not be able to reinforce the composite effectively. PALF, which remains long, can still effectively reinforce the composite [53]. The longer PALF also gives composites with higher impact strength and HDT (cf. Figure 8 and Figure 9). This observation supports our previous works, where PALF has been shown to outperform short Kevlar in reinforcing rubber matrices [22], and with an appropriate adhesion promoter the effectiveness can be further improved [54].

As stated above, all kinds of fiber are prone to breakage during compounding with polymer matrices due to different breakage mechanisms [55] depending on the fiber characteristics, and this includes PALF. It can be easily envisaged that by reducing the stress involved during compounding, either by increasing the temperature or reducing mixing speed, the breakage could be reduced or minimized. Mixing with a two-roll mill involves a much lower shear stress than with an internal mixer or screw extruder. It is clear from the results above that flax fiber still breaks, while PALF does not. The fact that PALF can maintain its length during mixing with thermoplastics and provide a high reinforcement efficiency certainly encourages its use in this form. Given that the starting length is long enough, and large fiber bundles break into finer elementary fibers during mixing, resulting in a significantly increased aspect ratio, composites with greatly improved properties can be obtained. Moreover, the utilization of PALF offers a promising ecological advantage. Compared to purposely cultivated fibers (such as flax, hemp, and kenaf), PALF exhibits lower carbon emissions and a reduced environmental footprint. Additionally, the use of PALF contributes to sustainable waste management practices by repurposing agricultural waste, making it a more environmentally friendly alternative for composite reinforcement. These ecological benefits further underscore the potential of PALF as a viable and eco-conscious solution in advancing sustainable materials across various industries, especially those that require higher performance or thinner parts.

## 5. Conclusions

In this study, we compared PALF with cultivated flax fiber as natural reinforcements in unidirectional PBS matrix composites. PALF showed remarkable potential as a sustainable alternative to flax fiber, well known for its high mechanical properties. PALF’s ability to maintain length and integrity during mixing led to significant improvements in the flexural strength and modulus, particularly at 20 wt.% fiber content. Successful PALF dispersion in the matrix, along with fiber bundle disintegration, resulting in higher aspect ratio, further contributed to its superior performance. PALF offers valuable ecological benefits, with a lower carbon footprint and the utilization of agricultural waste. The study highlights PALF’s underexplored potential as a sustainable and high-performance natural reinforcement, paving the way for eco-friendly materials in various industries. PALF’s effective reinforcement and ecological advantages suggest that it is a promising candidate for developing sustainable and eco-friendly materials.

## Figures and Tables

**Table 1:** Chemical composition of PALF and flax fibers.

Chemical Constituent (%)	PALF	Flax
Cellulose (%)	57.2	67.2
Holocellulose (%)	85.5	82.6
Lignin (acid soluble)	2.6	0.9
Lignin (acid insoluble)	7.8	6.5

**Table 2:** Thermal properties of PBS/PALF and PBS/flax composites.

Sample	First Heating		Cooling	Second Heating	
	*T*_m_ (°C)	*X*_c_ (%)	*T*_c_ (°C)	*T*_m_ (°C)	*X*_c_ (%)
PBS	115.0	75.4	84.4	114.4	72.6
10PALF	114.2	78.9	81.4	114.2	75.7
20PALF	114.4	79.0	82.8	114.0	75.5
10Flax	114.8	76.2	81.0	114.8	72.9
20Flax	115.0	76.4	81.8	115.3	72.6

## Data Availability

Not applicable.
